# Progress in Pediatrics

**Published:** 1904-03-19

**Authors:** 


### PROGRESS IN PEDIATRICS.

**Gonococcal Peritonitis.**—Dr. W. P. Northrup [1] records two cases of this affection in two sisters, aged respectively 9 and 11 years, who accidentally acquired vulvo-vaginitis, which developed a week later into peritonitis. There seems to be something characteristic about the abrupt onset of peritoneal symptoms in cases of gonococcal peritonitis, which may ensue even when the vulvo-vaginitis is under treatment. Pain and tenderness appear suddenly, and prostration and pallor are pronounced. Other signs present are pinched features, anxious expression, algidity, rapid thoracic respiration, and painful micturition and defæcation. Operation may appear urgently called for, as in one of the above cases, but is apparently of little therapeutic value. The peritoneum generally was found to be intensely injected, and there was a considerable amount of straw-coloured fluid in the pelvis. The prognosis would appear to be good. The condition should be suspected in female children who present vulvo-vaginitis with signs of peritonitis of unexplained origin. In Dr. Northrup's paper he says that four members of the family were infected with vulvo-vaginitis and urethritis, through accidental contamination, but only two developed peritonitis. The gonococcus was identified in all the cases. Although complete recovery may occur as regards the vulvo-vaginitis and peritonitis, it is not known whether the genital tracts may not be functionally impaired or destroyed by the inflammation.

**Acutely Occurring Deafness and Blindness.**—Dr. Alex. James [2] records the case of a girl of 13 who was suddenly seized with vomiting, headache, and fever. General tenderness was present and stiffness of the neck muscles. She became comatose, and remained so for some days, when improvement set in and was followed by complete recovery, save that persistent nerve deafness remained. Another case was that of a boy of 7 who became suddenly ill with high fever, severe headache, sickness, and vomiting. The acute symptoms gradually subsided, but the pupils became dilated and the boy became quite blind. Under treatment the blindness had become less pronounced, so that he was able to count fingers at a distance of 8 feet, but well-marked optic atrophy had supervened. Discussing these cases, Dr. James refers to Dr. Voltolini's view as to a certain affection of childhood which he terms labyrinthitis or otitis labyrinthica, and which runs a course similar to that described in the first case. Dr. James thinks it is impossible to regard all the phenomena as due to a purely labyrinthine lesion. He then refers to the view that cases like the above are due to a form of meningitis, presumably of the cerebro spinal type, and finds a difficulty in accepting this, because of the way in which individual nerves are picked out, and because of the signs of a nuclear involvement. We think, however, that the frequency with which typical cases of meningitis are followed by symmetrical lesions of individual nerves is of more value from a diagnostic point of view than the comparatively slight difficulty to which he refers. Dr. James is inclined to view the two cases as of toxæmic origin, the toxin having a selective affinity for the nuclei of origin of certain nerves, as happens in the case of anterior poliomyelitis.

**Asthma.**—Dr. Douglas Stanley[3] is of opinion that true asthma is infrequent in childhood. He points out that a spasmodic condition of breathing may occur during the course of an attack of pneumonia or bronchitis; but this is not true asthma. Nevertheless, real typical asthma may occur during childhood, of which he relates a case in a girl of 11. The asthmatic attacks occurred periodically, usually about 3 P.M. The breathing was laboured, expiration being particularly difficult and accompanied by loud cooing sounds, which could be heard at a considerable distance. One curious feature in this case was the rise of temperature, which appeared and disappeared with the attack, and lasted only a few hours. Its maximum varied from 102° to 104°. The greatest relief was obtained from the use of the liquid extract of grindelia robusta in 15-minim doses every quarter of an hour, for four or five doses. This not only cut short an attack, but, when given before an attack was expected, was usually successful in warding it off. In other cases, however, the writer did not find much benefit from grindelia. He does not think that iodide of potassium in large doses is well tolerated in children. In making a diagnosis in a case of spasmodic breathing, other conditions, such as enlarged tracheal glands, disease of the thymus gland and auto-intoxication from intestinal absorption must be borne in mind. Skin lesions, usually of the eczematous or urticarial type, are frequently associated with asthma, and in two of the author's cases ichthyosis was present.

[1] Arch. of Pediatrics, Dec. 1903. [2] Scott. Med. and Surg. Jour July 1903. [3] Birm. Med. Rev., Feb. 1903.

*(To be continued.)*

